# The mTOR/AP-1/VEGF signaling pathway regulates vascular endothelial cell growth

**DOI:** 10.18632/oncotarget.10756

**Published:** 2016-07-21

**Authors:** Shuo Wang, Jiawei Lu, Qingsheng You, Hua Huang, Yingying Chen, Kun Liu

**Affiliations:** ^1^ Medical College, Nantong University, Nantong, China; ^2^ Department of Cardiothoracic Surgery, Affiliated Hospital of Nantong University, Nantong, China; ^3^ Department of Pathology, Affiliated Hospital of Nantong University, Nantong, China; ^4^ Department of Immunology, Nantong University, Nantong, China

**Keywords:** mTOR, AP-1, VEGF, vascular endothelial cell, signaling pathway

## Abstract

Vascular restenosis is a common adverse event following percutaneous coronary intervention (PCI) and coronary artery bypass grafting (CABG). The atypical Ser/Thr protein kinase mammalian target of rapamycin (mTOR) plays an important role in cell differentiation and apoptosis. Vascular restenosis caused by excessive endothelial cell proliferation can be inhibited by local application of the mTOR inhibitor rapamycin (RAPA); however, RAPA can also suppress normal vascular endothelial cell growth by blocking mTOR/VEGF signaling, although the underlying mechanism is still unclear. Here, endogenous mTOR, AP-1, and VEGF were inhibited or overexpressed to investigate the mechanism underlying the effects of RAPA. Inhibition of AP-1 or mTOR with AP-1-siRNA or RAPA treatment respectively, decreased vascular endothelial cell proliferation, upregulation of AP-1 or mTOR increased cell proliferation, and VEGF overexpression increased, while RAPA-induced mTOR inhibition decreased vascular endothelial cell proliferation, the results indicate that combining mTOR downregulation and VEGF upregulation might both inhibit restenosis and maintain normal vascular endothelial cell growth after PCI or CABG, suggest the mTOR/AP-1/VEGF pathway might play a crucial role in regulating vascular endothelial cell growth.

## INTRODUCTION

The incidence of human coronary heart disease (CHD) has recently been increasing year by year [1]. Percutaneous coronary intervention (PCI) and coronary artery bypass grafting (CABG) are the most common treatments for coronary heart disease [2]. Restenosis of the bridging vein can be suppressed by inhibiting mammalian target of rapamycin (mTOR), an atypical serine/threonine protein kinase that plays an important role in the regulation of cell proliferation [3–5].

Rapamycin (RAPA), an inhibitor of mTOR, inhibits proliferation and migration and accelerates apoptosis in endothelial cells, and delays the formation of new intima on the surface of stents [6]. RAPA-eluting stents, which decrease the incidence of coronary restenosis [7], are widely used in the treatment of coronary artery disease. The highly-specific vascular endothelial growth factor (VEGF) promotes proliferation, angiogenesis, and extracellular matrix alterations and increases vascular permeability, and RAPA treatment downregulates VEGF expression in vascular endothelial cells [8]. However, the mechanisms by which mTOR in regulates VEGF expression in vascular endothelial cells is unclear. Additionally, in cancer cells, activator protein-1 (AP-1) binds to the VEGF promoter and activates VEGF transcription [9, 10]. In this study, we explored the mechanism by which mTOR and the mTOR/AP-1/VEGF pathway regulates vascular endothelial cell proliferation.

## RESULTS

### siRNA-mediated downregulation of AP-1 decreased AP-1 and VEGF, but not mTOR levels

**Figure 1:**
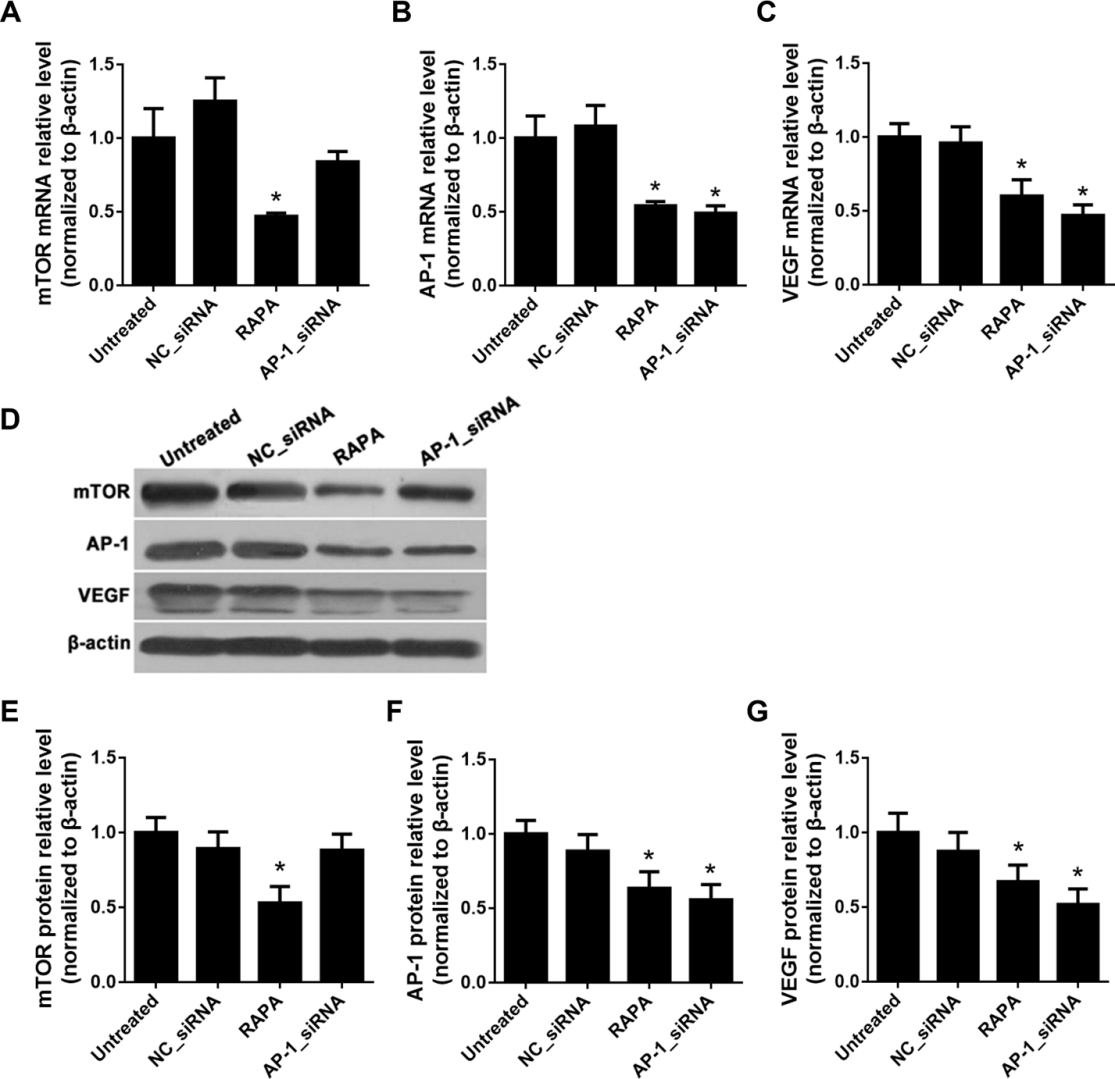
Effects of AP-1 downregulation on mTOR, AP-1, and VEGF expression (**A**) mTOR mRNA levels after treatment with AP-1-siRNA or RAPA were detected by RT-qPCR, *p < 0.05 vs. untreated cells. (**B**) AP-1 mRNA levels after treatment with AP-1-siRNA or RAPA were detected by RT-qPCR, *p < 0.05 vs. untreated cells. (**C**) VEGF mRNA levels after treatment with AP-1-siRNA or RAPA were detected by RT-qPCR, *p < 0.05 *vs.* untreated cells. (**D**) mTOR, AP-1, and VEGF protein levels after treatment with AP-1-siRNA or RAPA were detected by Western blot. (**E**) Relative mTOR protein levels, *p < 0.05 vs. untreated cells. (**F**) Relative AP-1 protein levels, *p < 0.05 vs. untreated cells. (**G**) Relative VEGF protein levels, *p < 0.05 vs. untreated cells.

RNA interference (RNAi) with an siRNA targeting AP-1 was used to suppress AP-1 expression, and subsequent changes in mTOR, AP-1, and VEGF mRNA and protein levels were detected using real-time quantitative PCR (RT-qPCR) and Western blot, respectively. Compared to untreated cells, RAPA, but not AP-1-siRNA, reduced mTOR mRNA and protein levels (*p* < 0.05) (Figure 1A, 1D, and 1E); both AP-1-siRNA and RAPA reduced AP-1 mRNA and protein levels (*p* < 0.05) (Figure 1B, 1D, and 1F). Both AP-1-siRNA and RAPA also reduced VEGF mRNA and protein levels (*p* < 0.05) (Figure 1C, 1D, and 1G). There were no differences in mTOR, AP-1, or VEGF levels between untreated and NC-siRNA-treated cells (*p* > 0.05).

### Effects of AP-1 or mTOR upregulation on mTOR, AP-1, and VEGF levels

**Figure 2:**
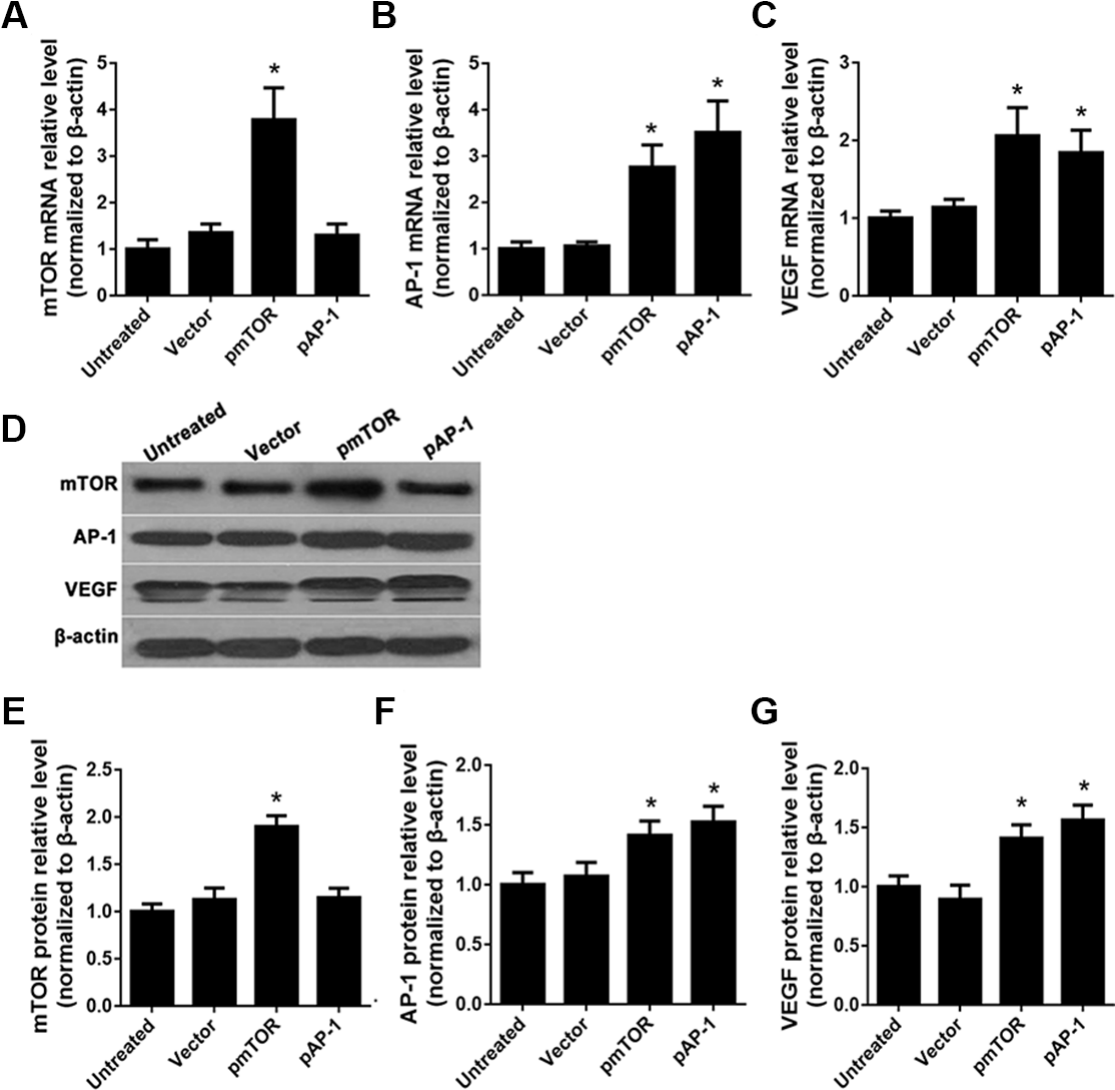
Effects of AP-1 and mTOR upregulation on mTOR, AP-1, and VEGF expression (**A**) mTOR mRNA levels after treatment with pAP-1 or pmTOR were detected by RT-qPCR, *p < 0.05 vs. untreated cells. (**B**) AP-1 mRNA levels after treatment with pAP-1 or pmTOR were detected by RT-qPCR, *p < 0.05 vs. untreated cells. (**C**) VEGF mRNA levels after treatment with pAP-1 or pm TOR were detected by RT-qPCR, *p < 0.05 vs. untreated cells. (**D**) mTOR, AP-1, and VEGF protein levels after treatment with pAP-1 or pmTOR were detected by Western blot. (**E**) Relative mTOR protein levels, *p < 0.05 vs. untreated cells. (**F**) Relative AP-1 protein levels, *p < 0.05 vs. untreated cells. (**G**) Relative VEGF protein levels, *p < 0.05 vs. untreated cells.

Overexpression vectors were constructed to upregulate endogenous gene expression in rat vascular endothelial cells (RAECs). Compared to untreated or empty vector control (vector)-treated cells, the mTOR overexpression vector (pmTOR) increased mTOR mRNA and protein levels (*p* < 0.05) (Figure 2A, 2D, and 2E). Both the AP-1 over-expression vector (pAP-1) and pmTOR increased AP-1 mRNA and protein levels (*p* < 0.05) (Figure 2B, 2D, and 2F). Both pAP-1 and pmTOR also increased VEGF mRNA and protein levels (*p* < 0.05) (Figure 2C, 2D, and 2G). There were no differences in mTOR mRNA or protein levels between pAP-1 and empty vector-treated cells (*p* > 0.05).

### AP-1 downregulation and RAPA decreased, while AP-1 and mTOR overexpression increased, proliferation in aortic endothelial cells

**Figure 3:**
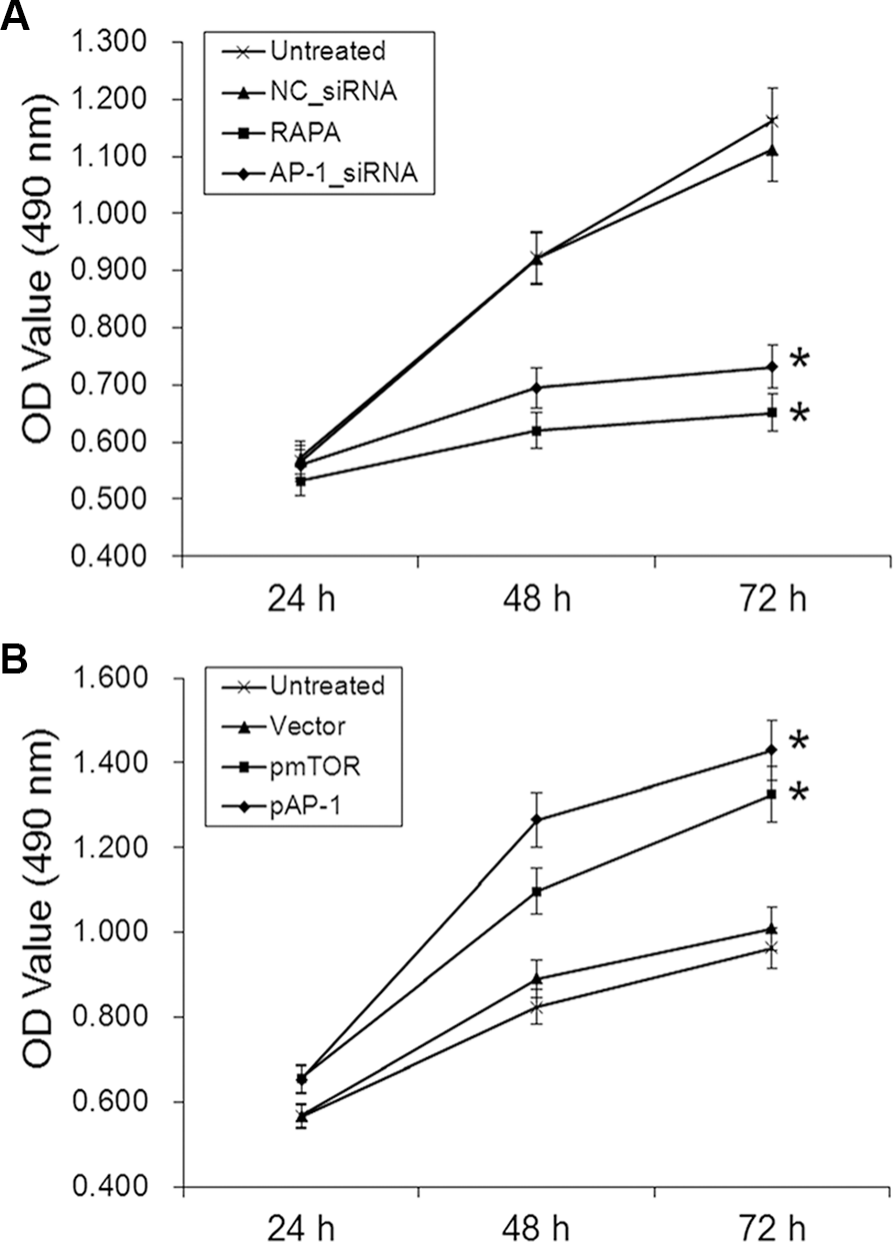
Effects of AP-1 or mTOR inhibition and overexpression on aortic endothelial cell proliferation (**A**) Cell growth curves for all groups 24, 48, and 72 h after AP-1-siRNA or RAPA treatment, *p < 0.05 vs. untreated cells. (**B**) Cell growth curves for all groups 24, 48, and 72 h after pmTOR or pAP-1 treatment, *p < 0.05 vs. untreated cells.

The proliferation of aortic endothelial cells treated with AP-1-siRNA or RAPA was measured using an MTT assay. Proliferation decreased in cells 48 and 72 h after treatment with AP-1-siRNA or RAPA compared to untreated or NC-siRNA-treated cells (*p* < 0.05, Figure 3A); there was no difference in proliferation between untreated and NC-siRNA-treated cells (*p* > 0.05). In contrast, proliferation increased in cells 48 and 72 h after treatment with pAP-1 or pmTOR compared to untreated or NC-siRNA-treated cells (*p* < 0.05, Figure 3B). Proliferation did not differ between untreated and empty vector-treated cells (*p* > 0.05).

### Effects of RAPA and pVEGF on mTOR, AP-1, and VEGF levels

**Figure 4:**
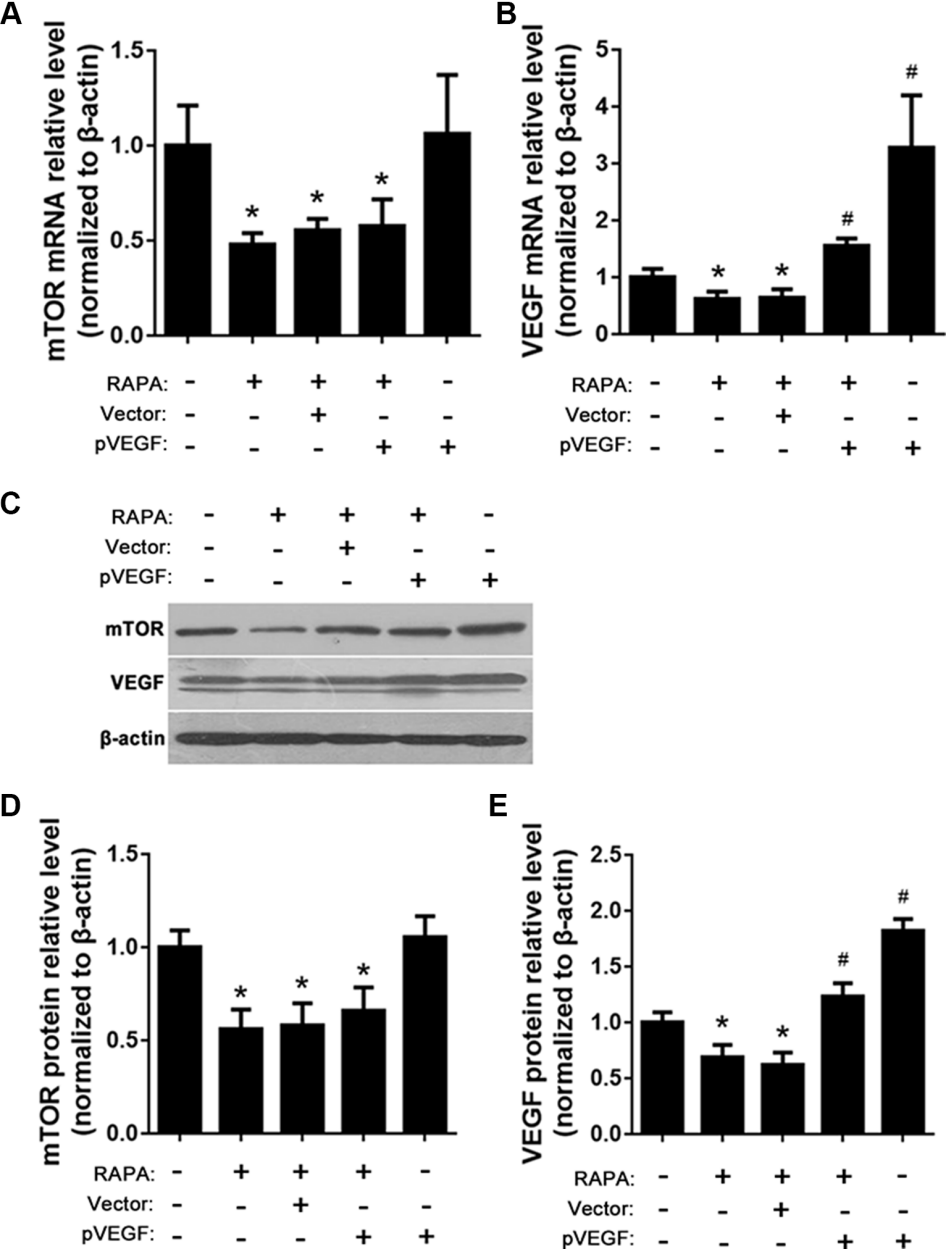
Effects of mTOR inhibition and VEGF overexpression on mTOR, AP-1, and VEGF expression (**A**) mTOR mRNA levels after RAPA and/or pVEGF treatment were detected by RT-qPCR, *p < 0.05 vs. untreated cells. (**B**) VEGF mRNA levels after RAPA and/or pVEGF treatment were detected by RT-qPCR, *p < 0.05 VEGF low expression vs. untreated cells, ^#^*p* < 0.05 VEGF high expression vs. untreated cells. (**C**) mTOR and VEGF protein levels after RAPA and/or pVEGF treatment were detected by Western blot. (**D**) Relative mTOR protein levels, *p < 0.05 vs. untreated cells. (**E**) Relative VEGF protein levels, *p < 0.05 VEGF low expression vs. untreated cells, ^#^*p* < 0.05 VEGF high expression vs. untreated cells.

mTOR and VEGF mRNA and protein levels were determined using RT-qPCR and Western blot, respectively, after treatment with pVEGF and/or RAPA. Compared to untreated cells, RAPA alone, empty vector with RAPA, and VEGF overexpression (pVEGF) with RAPA all reduced mTOR mRNA and protein levels (*p* < 0.05) (Figure 4A, 4C, and 4D). RAPA alone or with empty vector also reduced, while pVEGF alone or with RAPA increased, VEGF mRNA and protein levels (all *p* < 0.05); pVEGF alone increased VEGF levels more than combined treatment with pVEGF and RAPA (*p* < 0.05) (Figure 4B, 4C, and 4E).

### VEGF overexpression increased, and RAPA decreased, proliferation in aortic endothelial cells

Proliferation of aortic endothelial cells treated with RAPA and/or VEGF overexpression vector was measured in an MTT assay. Proliferation increased in cells 48, 72, and 96 h after treatment with pVEGF compared to untreated cells (*p* < 0.05). In contrast, proliferation decreased in cells 48, 72, and 96 h after treatment with RAPA either alone or combined with empty vector compared to untreated cells (*p* < 0.05). Proliferation did not differ between untreated cells and cells treated with a combination of pVEGF and RAPA (*p* > 0.05).

## DISCUSSION

Mammalian target of rapamycin (mTOR) is an atypical Ser/Thr protein kinase that interacts with multiple growth factors and cytokines and participates in different signaling pathways to affect transcription and protein synthesis. mTOR, which is a downstream molecule of the PI3K/Akt/mTOR signaling pathway, is inhibited by rapamycin (RAPA). Vascular restenosis resulting from percutaneous coronary intervention (PCI) or coronary artery bypass grafting (CABG), as well as vascular endothelial cell growth, is inhibited by RAPA-induced mTOR suppression [11]. Vascular restenosis after PCI or CABG reduces survival rates and quality of life in patients with coronary heart disease, and vascular smooth muscle cell proliferation is a major cause of restenosis. Therefore, coronary heart disease patients are usually treated with RAPA to inhibit the proliferation and migration of vascular smooth muscle cells and thus prevent restenosis [12].

VEGF is a key growth factor that promotes vascular endothelial cell proliferation. RAPA inhibits VEGF expression in aortic endothelial cells and likely contributes to thrombosis in coronary artery disease patients implanted with RAPA-eluting stents [13]. Hudson *et* *al.* [14] found that RAPA-induced inhibition of HIF-1 alpha downregulated VEGF, resulting in an inhibition of tumor angiogenesis. However, the mechanism by which mTOR regulates VEGF is unclear. AP-1, an intracellular transcription activator, binds to the promoter region of the VEGF gene [15] and upregulates VEGF expression in many cancers, including head and neck cancer [16], breast cancer [17], colon carcinoma [18], and ovarian carcinoma [19]. In order to clarify the role of the mTOR signaling pathway in vascular endothelial cell proliferation, we used siRNA and overexpression vectors to down- or upregulate AP-1, mTOR, and VEGF in rat vascular endothelial cells (RAEC) and measured resulting changes in mRNA expression, protein levels, and cell proliferation.

**Figure 5:**
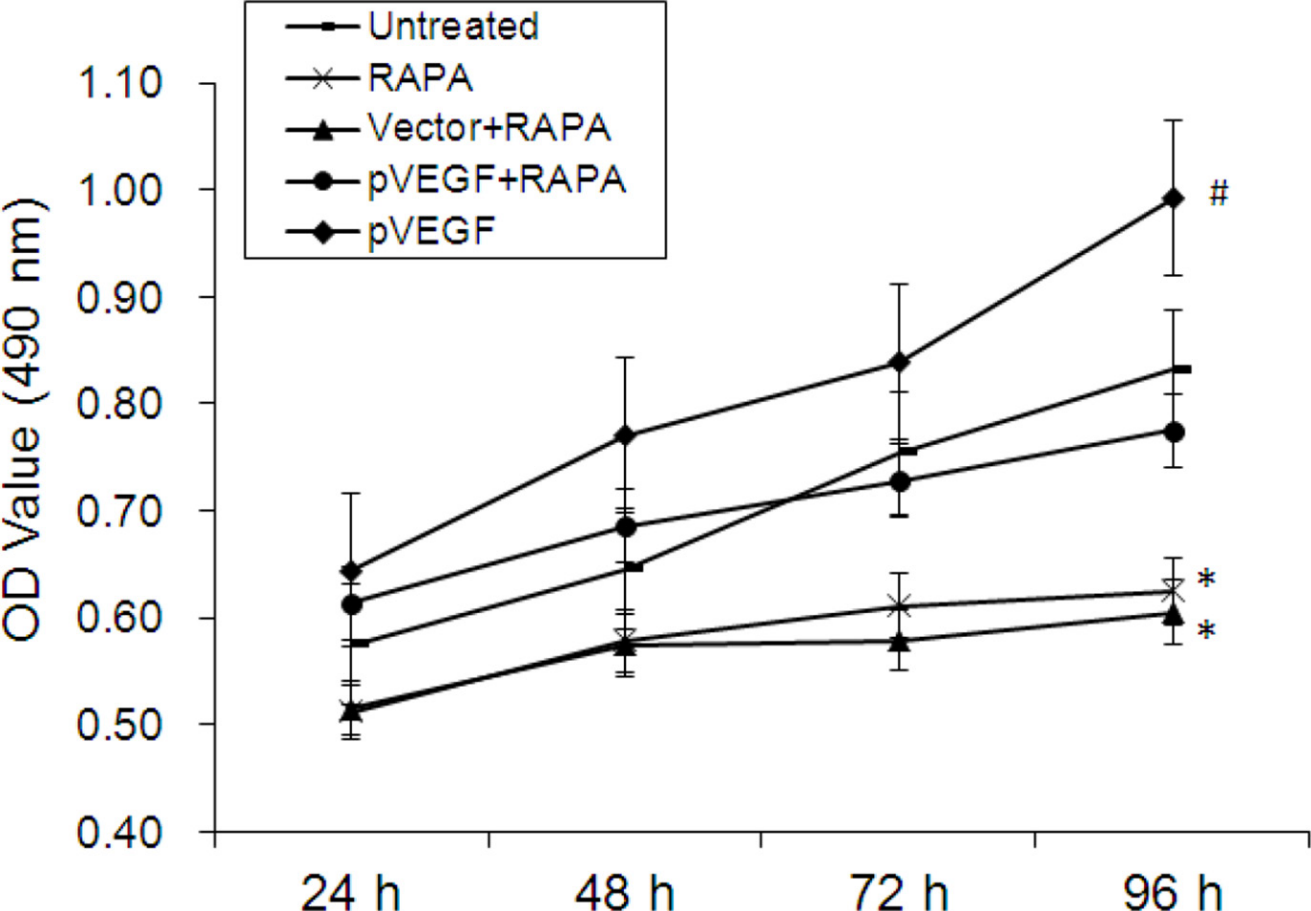
Effects of mTOR inhibition and VEGF overexpression on aortic endothelial cell proliferation Cell growth curves for all groups 24, 48, 72, and 96 h after treatment, *p < 0.05 pVEGF vs. untreated cells, ^#^*p* < 0.05, RAPA or empty vector combined with RAPA (vector+RAPA) vs. untreated cells. Proliferation did not differ between untreated cells and cells treated with pVEGF combined with RAPA (pVEGF+RAPA) (*p* > 0.05).

**Figure 6:**
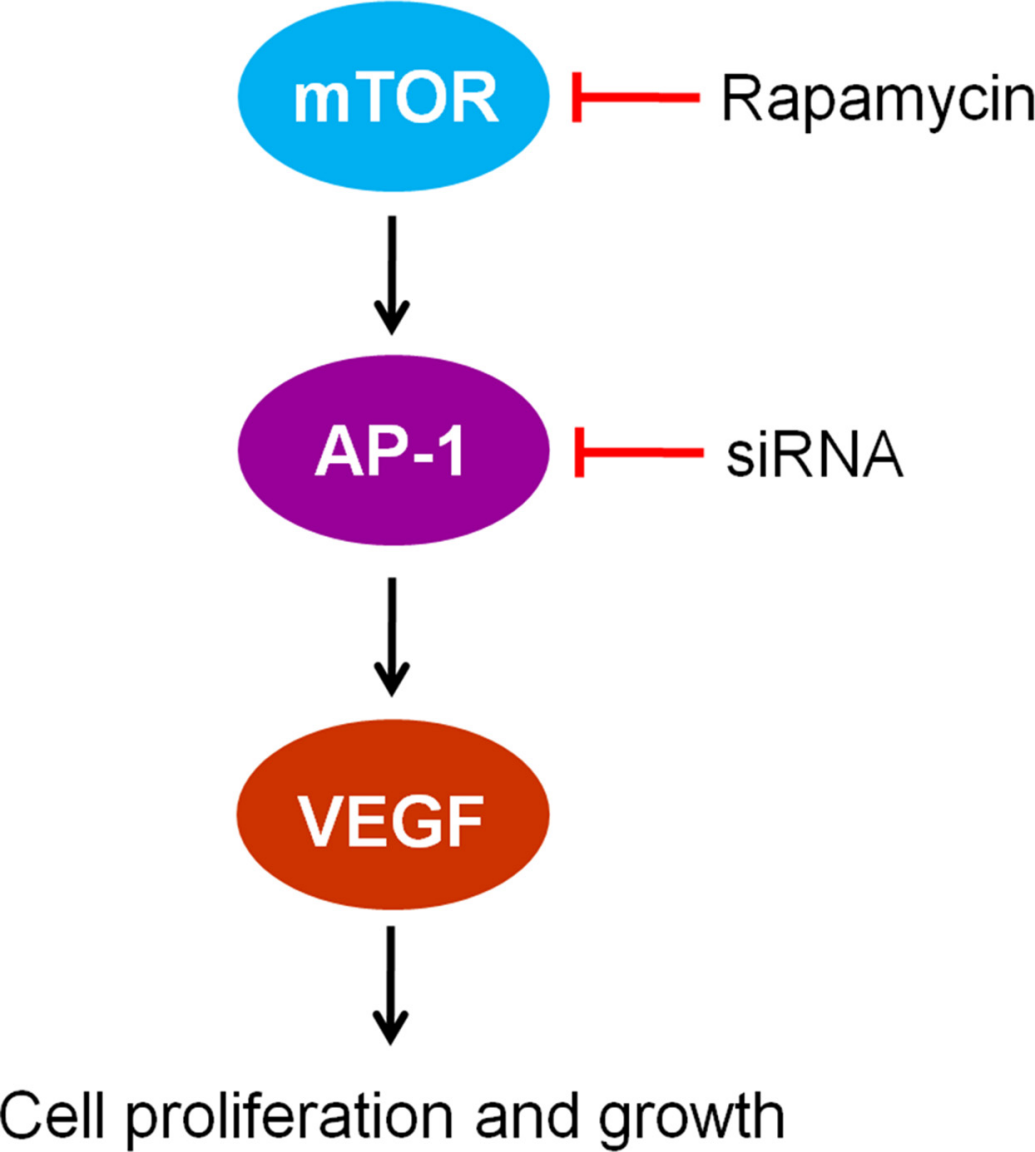
The mTOR/AP-1/VEGF signaling pathway regulates vascular endothelial cell growth

We found that RAPA treatment reduced mTOR, AP-1, and VEGF mRNA and protein levels in RAECs. siRNA-mediated AP-1 knockdown (AP-1-siRNA) also reduced VEGF mRNA and protein levels. RAPA-induced mTOR inhibition reduced both AP-1 and VEGF levels (Figure 1). Furthermore, overexpression of mTOR (pmTOR) upregulated mTOR, AP-1, and VEGF, while overexpression of AP-1 (pAP-1) upregulated AP-1 and VEGF (Figure 2). These results suggest that mTOR may act via AP-1 to regulate VEGF expression. We then used MTT assays to determine whether RAEC proliferation was regulated by mTOR, AP-1, or VEGF. Inhibition of AP-1 or mTOR with AP-1-siRNA or RAPA treatment, respectively, decreased RAEC proliferation (Figure 3B). Conversely, upregulation of AP-1 or mTOR increased cell proliferation (Figure 3B). Moreover, overexpression of VEGF combined with RAPA treatment inhibited mTOR expression, and RAPA alone or in combination with empty vector inhibited VEGF expression. In contrast, overexpression of VEGF alone, or to a lesser degree in combination with RAPA, increased VEGF expression (Figure 4). Finally, VEGF overexpression increased, while RAPA-induced mTOR inhibition decreased aortic endothelial cell proliferation; proliferation did not differ between untreated cells and VEGF-overexpressing cells treated with RAPA (Figure 5). Together, these results indicate that combining mTOR downregulation and VEGF upregulation might both inhibit restenosis and maintain normal vascular endothelial cell growth after PCI or CABG. Furthermore, the mTOR/AP-1/VEGF pathway may play a crucial role in the regulation of vascular endothelial cell growth (Figure 6).

## MATERIALS AND METHODS

### Cell lines and cell culture

Rat aortic endothelial cells (RAEC) were obtained from the Cell Center of Central South University (China), cultured in Dulbecco's Modified Eagle Medium (DMEM) (Gibco, USA) supplemented with 10% fetal bovine serum (FBS) (Gibco, USA), and grown in a 37°C, 5% CO_2_ incubator with 95% humidity.

### Transfection of siRNA and plasmid DNA

The sequences of siRNA targeting AP-1 (AP-1-siRNA) were as follows: sense 5′-UGUAGUGGUG AUGUGCCCAUUGCUGdTdT-3′, antisense 5′-CAGCA AUGGGCACAUCACCACUACAdTdT-3′. Rat AP-1, mTOR, and VEGF cDNA were all cloned into pEGFP-N3 vectors (Clontech, USA) to generate pAP-1, pmTOR, and pVEGF overexpression vectors; empty pEGFP-N3 was used as a vector control. The siRNA and plasmids were all designed or constructed by Biomics Biotechnologies Co., Ltd. (China). RAECs were transferred to cell culture plates and grown until they reached the logarithmic growth phase; when cells reached to 50 to 70% confluence, they were transfected with siRNA or plasmids using Lipofectamine^®^ 2000 (Thermo Fisher, USA) according to the manufacturer's instructions. All applicable groups were treated with 5 nM rapamycin (RAPA).

### Real-time quantitative PCR (RT-qPCR)

RNA isolation was performed using TRIzol Reagent (Thermo Fisher, USA). PCR reactions were then performed. 12.5 μL of 2 × OneStep Master Mix (Biomics Biotech, China), 0.5 μL each of forward and reverse primers (10 μM each, Biomics Biotech China), 0.5 μL of 50 × SYBR Green I, and 4 μL total RNA were added to each reaction, and reverse transcription was performed at 42°C for 30 min and 85°C for 5 min. PCR consisted of denaturation at 95°C for 10 min followed by 45 cycles of 95°C for 20 sec, 55°C for 30 sec, 72°C for 30 sec, 95°C for 1 min, and 55°C for 30 sec. Rat β-actin served as an internal control. All primer sequences are shown in Table 1. The experiment was performed in triplicate. The results were analyzed using the 2^-ΔΔCt^ method [20].

**Table 1:** RT-qPCR primer sequences

Gene Name		Sequence (5′–3′)	Amplicon Length
mTOR	F	CACATCACTCCCTTCACCA	153 bp
	R	GCAACAACGGCTTTCCAC	
VEGF	F	CCTCGTGGAACTGGATTCG	109 bp
	R	TATGTGGGTGGGTGTGTCTA	
AP-1	F	AAGAACACAAAGCAGGGAGG	75 bp
	R	GGGAGTTCATCCGCAATCTA	
β-actin	F	GGGAAATCGTGCGTGACATT	76 bp
	R	GCGGCAGTGGCCATCTC	

F: Forward primer, R: Reverse primer.

### Western blotting

RAECs treated as described above were washed twice with ice-cold PBS and lysed in SDS protein lysis buffer (Pierce, USA). Protein levels were detected using the BCA protein assay kit (Pierce, USA). 20 μg of total cell lysate were loaded and separated by electrophoresis on SDS acrylamide gels, transferred onto a PVDF membrane, and blocked in 5% milk in TBST (20 mM Tris, 150 mM NaCl, 0.05% Tween-20, pH 7.5) for 2 h. Blots were then incubated overnight at 4ºC with the following primary antibodies: rabbit anti-rat AP-1 polyclonal antibody (Proteintech, USA, 1:500 dilution), rabbit anti-rat VEGF polyclonal antibody (Proteintech, USA, 1:500 dilution), rabbit anti-human mTOR polyclonal antibody (Proteintech, USA, 1:500 dilution), and mouse anti-rat β-actin monoclonal antibodies (Boster, China, 1:400 dilution). Membranes were then washed three times with TBST and incubated with goat anti-rabbit (Santa Cruz, USA, 1:500 dilution) or goat anti-mouse (Santa Cruz, USA, 1:500 dilution) secondary antibodies as appropriate. The optical density of the imprinted region was determined using AlphaImager (FluorChem5500; Alpha Innotech, USA).

### Cell proliferation assay

RAECs proliferation was measured with an MTT assay. RAECs were seed in a 96-well plate (1 × 10^4^ cells/mL) and treated as described above for 0, 24, 48, 72, or 96 h. Ten μL of MTT (5 mg/mL; Promega, USA) was then added to each well followed by incubation at 37°C protected from light for 4 h. After incubation, MTT was removed and 100 μL of DMSO was added to each well. The absorbance value of each well was measured at a wavelength of 490 nm with a Microplate Reader (Bio-Rad, USA). All proliferation assays were performed in triplicate.

### Statistical analysis

Statistical analyses were conducted with SPSS19.0. All the data are shown as mean ± SEM of at least three independent experiments. Significance was determined using one-way ANOVAs followed by post hoc tests; *p* < 0.05 was considered statically significant.
